# Crop–livestock integration enhanced soil aggregate-associated carbon and nitrogen, and phospholipid fatty acid

**DOI:** 10.1038/s41598-022-06560-6

**Published:** 2022-02-17

**Authors:** Sangeeta Bansal, Poulamee Chakraborty, Sandeep Kumar

**Affiliations:** Department of Agronomy, Horticulture, and Plant Science, South Dakota State University, Brookings, SD 57007 USA

**Keywords:** Agroecology, Grassland ecology, Microbial ecology

## Abstract

Integrated crop–livestock (ICL) production enhances diversification and provides ecosystem benefits by improving nutrient cycling and energy efficiency, thus, increasing overall farm productivity. However, a detailed study is needed to understand the influence of crop diversification and grazing animals on soil aggregation and associated carbon (C) and nitrogen (N), and microbial properties, especially compared with a grazed native pasture. We investigated the soil aggregate size distribution and associated C and N fractions, glomalin-related soil protein, and soil phospholipid fatty acid (PLFA) to understand the collective influence of livestock grazing of crop residue and cover crops (CC) and compared it with native pasture and non-grazed traditional production systems. The study was conducted in South Dakota at four different locations consisting of three long-term (> 30 years) on-farm sites: 1 (Salem), 2 (Bristol), 3 (Bristol) with three treatments that included ICL (corn, *Zea Mays* L.-soybean, *Glycine max* L.-oats, *Avena sativa* L.-CC with cattle grazing); natural ecosystem (NE) or native pasture; and control (CNT) (corn–soybean-without grazing or CC). Experimental site 4 (Beresford) with study duration of 3-year consisted of oats, oats with CC, oats with CC + grazing, and grazed pasture mix. Soil samples were collected from 0 to 5 cm depth at all four sites in summer 2019. Data showed that at sites 1 and 2, ICL had significantly (*P* ≤ 0.5) greater fractionation of 0.053–0.25 mm and > 4 mm aggregates compared with NE and CNT. At site 1, ICL showed significantly higher soil organic carbon (SOC, 36–49%) and higher nitrogen (33–44%) in > 4 mm aggregates than NE and CNT. At site 2, ICL had 32–41% higher SOC than NE and CNT for 0.25–0.5 mm aggregates. At site 1, NE enhanced total phospholipid fatty acid (PLFA), total bacterial biomass, gram (+), gram (−) bacteria than CNT, however, it did not vary significantly than ICL. Grazed pasture mix at site 4 had higher total PLFA (40.81 nmol g^−1^ soil) than the other treatments. The principal components 1 and 2 accounted for 33% and 22% of the variation, respectively, where the majority of the microbial compositions and aggregate-associated C and N were influenced by ICL and NE compared with corn–soybean without grazing or short-term oats/CC/grazing treatments. Integrated crop–livestock system and NE enhanced C and N concentrations in macroaggregates as well as in microaggregates. It is concluded that ICL and NE systems are sustainable prospects in enhancing overall soil health. Integrating crop and livestock improved physicochemical and microbial properties compared to the traditional corn–soybean system.

## Introduction

Integration of livestock in a crop production system intensifies crop yield and soil quality by leveraging synergies between multiple agroecosystem components and the added complexity due to trophic level (i.e. grazing animals) rather than relying on the increased conventional input levels such as fertilizers^1^. Apart from facilitating diversification causing environmental and soil health benefits, integrated crop–livestock (ICL) systems may enhance overall farm profitability^2,3^, reduce weed and pest^4^, improve nutrient cycling as a result of rotating livestock, and avoid the accumulation of detrimental nutrient levels from animal manure^2,5^. Previously an increase in corn (*Zea mays* L.) yield has been observed where winter cover crop (CC) was cattle grazed before the spring planting of corn as compared to continuous corn^6^.

Integrated crop–livestock systems can influence soil biological, physical, and chemical properties^7,8^. Previous ICL studies evidenced increased total nitrogen (N), carbon (C) sequestration, soil microbial biomass C, and a decrease in penetration resistance, which are associated with enhanced stable aggregate fraction^6,9–12^. It is observed that the inclusion of cover crops or manure application can alter the microbial composition in soil, influencing the C and N cycling and soil aggregate formation^13^. In addition, aggregate size determines the stability of SOC and N, e.g., decrease in macroaggregate turnover time stabilizes C, enhancing the formation of microaggregates aiding in long-term C sequestration^14^. Thus, it is important to understand the distribution of aggregates in soil and the associated C and N concentrations. Soil microbial community assessment is a great option because it easily responds to shifts in soil due to management systems than the other soil parameters^15,16^. Moreover, soil microorganisms perform a critical role in C cycling and can considerably affect the C accumulation in soil organic matter^17^. Grazing systems can depict conflicting responses of soil microbial communities due to several factors associated with intrinsic soil properties, environmental factors, and grazing intensity^18,19^.

Livestock has traditionally grazed the Northern Great Plains grasslands; however, these grasslands have been converted to cropland, consequently impacting the ecosystem services. Between 1997 and 2007, the Northern Plains States accounted for 57% of U.S. rangeland to cropland conversion, an estimated total of > 770,000 acres and approximately 1.1% of 1997 rangeland acreage^20^. Based on the research conducted at various sites in the U.S., a generalized solution is provided suggesting that annual cropping systems can replace forage lost through land conversion and enhance ecosystem benefits. However, to implement these strategies on farms throughout the Great Plains, producers’ participation is critical to promote diversified farm production, improve food security, and increase ecosystem resilience. Therefore, this study includes native grasslands of > 50 years at producers’ farms, part of which was converted to croplands by the producer. This study also includes short-term (~ 3 years) oats planted with cover crops and grazing, and grazed pasture mix treatments to assess whether aggregate formation or associated C and N fractions or microbial properties are responding to the multifaceted grazing management practices. Often changes in soil parameters especially related to C and N occur gradually and therefore subtle changes are left indistinguishable in the short term. Soil microbial biomass has previously been shown to be sensitive to short-term changes in soil management^21^. The short-term effects of management on SOC fractions and microbial community composition are complex and depend on the climatic region, soil type, crop sequence and residue returned^22^.

Ecosystem processes under cattle grazed systems in the field such as greater nutrient cycling^23^, higher microbial functional diversity^10^ and improved soil structure and organic matter^1^ indicate increased biophysical protection to less favorable ecological conditions, that require additional evaluation and comparison with the native grassland systems^1,24^. However, if the idea is to utilize ICL systems as a tool for sustainable growth, it is important to evaluate its contribution to a productive system as well as long-term stability. Previous studies have shown positive^2,9^ and negative^25–27^ responses of ICL system to soil health parameters. These responses among studies can be a result of different climatic conditions, historical land use, cropping practices, and region-specific ICL outcomes over time^11,28^. It is understood that the conservation practices can improve parameters related to soil health^29,30^, however, the effect of increasing system diversity along with the addition of ecological complexity by added cattle grazing on soil aggregation and associated C and N, and microbial composition need to be studied.

This study was conducted to test the hypothesis that the addition of livestock grazing and cover crop in a system can positively influence soil biological properties, aggregation and aggregate-associated C and N compared to traditional corn–soybean (*Glycine max*. L) rotation, laid out as a control. In terms of study duration, it is hypothesized that short-term grazing and cover crops (< 3-years) may not possibly influence these soil properties. The objectives of this study were to evaluate the shared influence of livestock grazing and cover crops on (i) aggregate size distribution (ii) aggregate-associated C and N fractions (iii) glomalin-related soil protein (iv) soil phospholipid fatty acid (PLFA) for microbial composition (vii) relationship between soil aggregation, C and N fractions, glomalin-related soil protein and PLFA under three long-term producers’ sites (> 30 years) and one short-term experimental site (3 years) in South Dakota. These parameters were also compared to investigate whether the ICL system can prove as beneficial as a long-term native grazed pasture system in terms of improving soil C and N fractions and microbial biomarkers.

## Results

### Aggregate size distribution

Significant differences in ICL, NE, and CNT were observed among aggregate size distributions at sites 1, 2, and 3, however, at site 4, only 1–2 and 2–4 mm aggregates responded to the grazed pasture, oats, and CC treatments (Table 1). Integrated crop–livestock system at sites 1 and 2 resulted in a significantly (*P* ≤ 0.5) greater fractionation of > 4 mm aggregates than the NE and CNT. Moreover, ICL enhanced 0.053–0.25 mm aggregates at all three sites compared with NE and CNT. A higher proportion of 1–2 mm and 2–4 mm aggregates was observed under grazed pasture mix than the oats, oats + CC, and oats + CC + grazing treatments at site 4.

**Table 1:** Aggregate size distribution at 0–5 cm soil depth under different production systems at four different sites.

Treatments	Aggregate size distribution (g 100 g^−1^ soil)					
	Aggregate size (mm)					
	> 4	2–4	1–2	0.5–1	0.25–0.5	0.053–0.25
**Site 1**						
ICL^§^	50.1^a†^	3.66^b^	3.90^b^	7.85^a^	1.69^a^	19.3^a^
NE	40.1^b^	24.1^a^	6.40^a^	7.22^a^	1.81^a^	11.2^c^
CNT	38.3^b^	3.94^b^	3.80^b^	8.79^a^	0.87^b^	15.5^b^
**Site 2**						
ICL	39.3^a^	4.50^b^	3.52^b^	7.41^b^	16.3^b^	11.8^a^
NE	32.6^b^	13.6^a^	11.1^a^	7.37^b^	20.5^b^	6.35^b^
CNT	31.1^b^	2.90^b^	4.97^b^	12.9^a^	28.2^a^	2.41^c^
**Site 3**						
ICL	40.8^a^	12.9^a^	10.6^a^	10.2^b^	12.5^b^	11.1^a^
NE	24.2^b^	11.5^a^	12.4^a^	14.2^a^	18.1^a^	6.59^b^
CNT	33.8^a^	5.86^b^	5.06^b^	11.1^ab^	20.1^a^	3.61^c^
**Site 4**						
Oat	42.2^a^	4.09^b^	2.38^b^	3.83^a^	7.74^a^	14.7^a^
Oat w/cc	32.2^a^	3.88^b^	3.60^b^	6.79^a^	9.43^a^	17.6^a^
Oat w/cc + g	43.1^a^	4.84^b^	2.93^b^	4.11^a^	6.76^a^	14.5^a^
Grazed pasture mix	34.4^a^	11.1^a^	7.55^a^	7.17^a^	7.44^a^	13.4^a^
**ANOVA (*P* > *F*)**						
Site 1: Treatments	0.0207	< 0.0001	0.0081	0.3727	0.0181	< 0.0001
Site 2: Treatments	0.0060	0.0019	< 0.0001	0.0057	0.0066	< 0.0001
Site 3: Treatments	0.0064	0.0111	0.0023	0.0492	0.0062	< 0.0001
Site 4: Treatments	0.8291	0.0051	0.0217	0.2265	0.8405	0.8562

^§^*ICL* integrated crop–livestock, *NE* natural ecosystem, *CNT* control, *CC* cover crop, *g* grazing.

^†^Means followed by different letters within a column under each site are significantly different at *P* ≤ 0.05 according to Fisher’s LSD.

Integrated crop–livestock system and NE enhanced (*P* ≤ 0.05) MWD at site 1 by 28% and 29%, respectively, compared with CNT. Similarly, for site 2, average MWD was significantly greater for ICL and NE systems compared with CNT (Table 2). However, at site 3, ICL system showed 44% and 28% significantly higher MWD than NE and CNT, respectively. Natural ecosystem resulted in higher GMD than ICL and CNT at site 1, however, it did not differ significantly from ICL at site 2. Conversely, NE showed significantly lower GMD than ICL at site 3. The MWD and GMD did not vary statistically among any treatments at site 4. Integrated crop–livestock resulted in significantly higher (~ 47%) glomalin related soil protein than CNT at site 1 (Table 2). However, no statistical difference was observed among any treatments at the rest of the sites.

**Table 2:** Mean weight diameter (MWD), geometric mean diameter (GMD), and glomalin at 0–5 cm soil depth as influenced by different production systems at sites 1, 2, 3, and 4.

Treatments	MWD (mm)	GMD (mm)	Glomalin (mg g^−1^ soil)
**Site 1**			
ICL^§^	3.37^a†^	0.24^b^	5.13^a^
NE	3.31^a^	0.33^a^	4.74^ab^
CNT	2.56^b^	0.18^b^	3.48^b^
**Site 2**			
ICL	2.68^a^	0.16^ab^	4.65^a^
NE	2.67^a^	0.19^a^	4.23^a^
CNT	2.24^b^	0.11^b^	3.70^a^
**Site 3**			
ICL	3.13^a^	0.24^a^	6.49^a^
NE	2.17^b^	0.12^b^	5.49^a^
CNT	2.44^b^	0.17^b^	6.11^a^
**Site 4**			
Oat	2.77^a^	0.19^a^	4.17^a^
Oat w/cc	2.22^a^	0.08^a^	5.06^a^
Oat w/cc + g	2.85^a^	0.21^a^	4.53^a^
Grazed pasture mix	2.61^a^	0.18^a^	4.59^a^
**ANOVA (*P* > *F*)**			
Site 1: Treatments	0.0117	0.0032	0.0435
Site 2: Treatments	0.0128	0.0479	0.3125
Site 3: Treatments	0.0056	0.0050	0.2264
Site 4: Treatments	0.8620	0.8548	0.8050

^§^*ICL* integrated crop–livestock, *NE* natural ecosystem, *CNT* control, *CC* cover crop, *g* grazing.

^†^Means followed by different letters within a column under each site are significantly different at *P* ≤ 0.05 according to Fisher’s LSD.

### Aggregate-associated carbon and nitrogen

**Figure 1:**
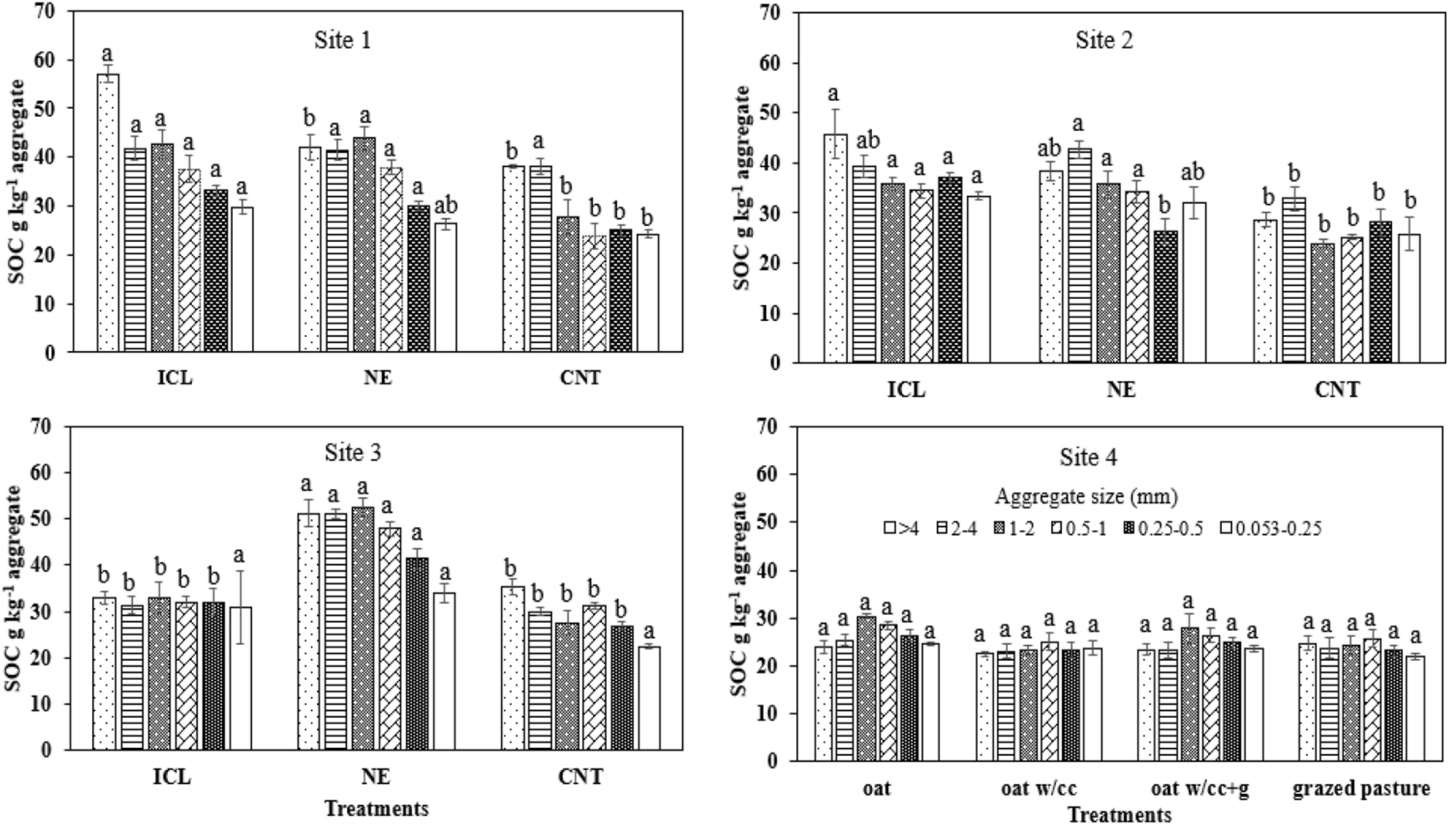
Soil aggregate-associated organic carbon concentration at 0–5 cm depth as influenced by different production systems at sites 1, 2, 3, and 4. Under each site, bars followed by different letters within an aggregate size at different treatments are significantly different at *P* ≤ 0.05 according to Fisher’s LSD. *ICL* integrated crop–livestock, *NE* natural ecosystem, *CNT* control, *CC* cover crop, *g* grazing.

Integrated crop–livestock system at site 1 showed a significantly higher SOC in > 4 mm aggregates with mean value of 57.1 g kg^−1^ aggregate, which was 36% and 49% greater than NE and CNT treatments, respectively (Fig. 1). For the aggregates 1–2, 0.5–1, 0.25–0.5 mm, ICL and NE showed significantly higher C than the CNT at site 1. Though at site 2, SOC did not vary significantly between ICL and NE treatments for aggregates > 4, 2–4, 1–2, 0.5–1, and 0.053–0.25 mm sizes, however, ICL had ~ 41% higher SOC than NE for 0.25–0.5 mm aggregates. At site 3, NE treatment performed considerably better than ICL and CNT for aggregates ranging between 0.25 to > 4 mm. Oats, CC, and grazing treatments at site 4 did not reveal any significant differences (*P* ≥ 0.05) in aggregate-associated SOC.

**Figure 2:**
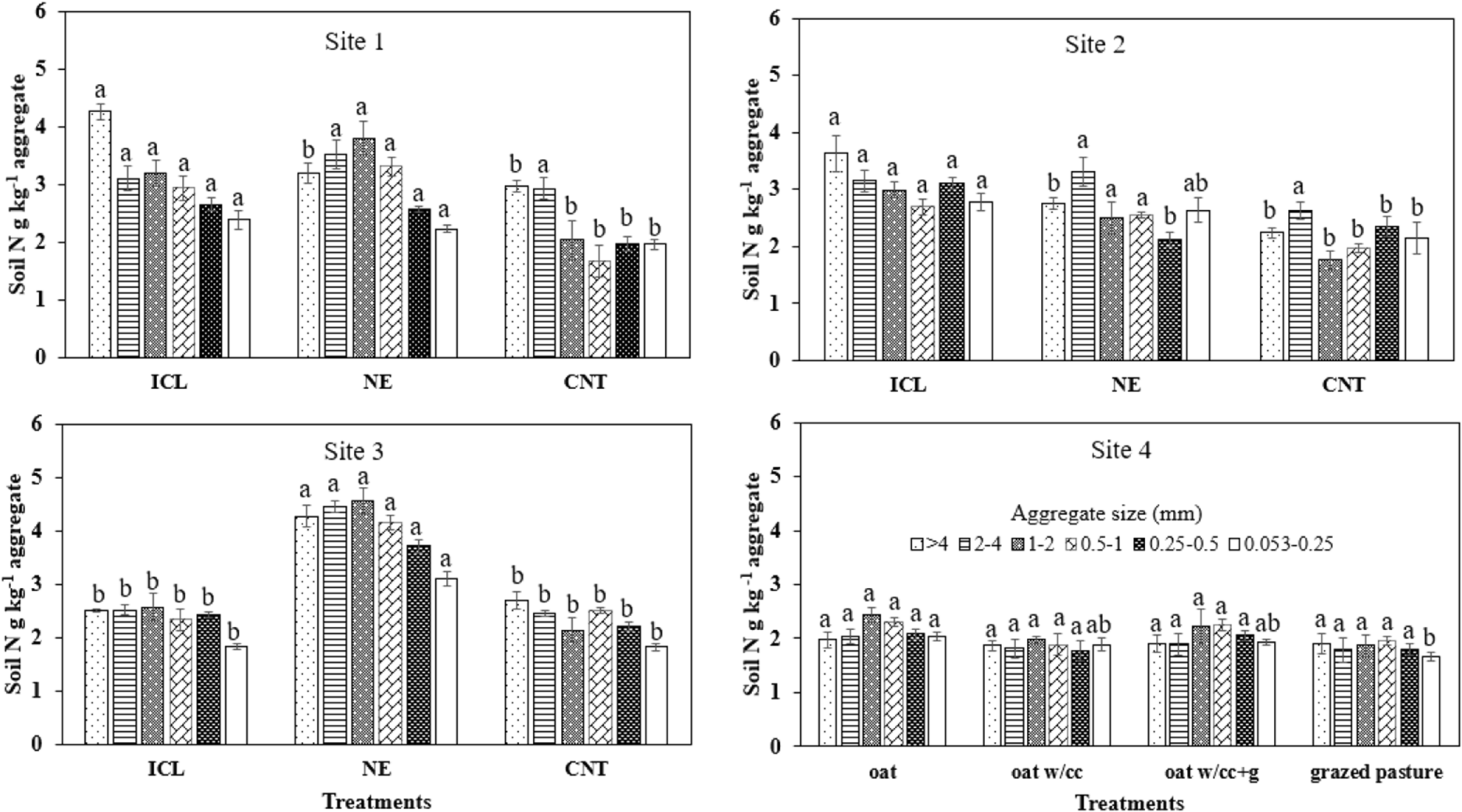
Soil aggregate-associated nitrogen concentration at 0–5 cm depth as influenced by different production systems at sites 1, 2, 3, and 4. Under each site, bars followed by different letters within an aggregate size at different treatments are significantly different at *P* ≤ 0.05 according to Fisher’s LSD. *ICL* integrated crop–livestock, *NE* natural ecosystem, *CNT* control, *CC* cover crop, *g* grazing.

At site 1, ICL system showed 33–44% higher N than NE and CNT for > 4 mm aggregates, whereas for aggregates 1–2, 0.5–1, 0.25–0.5, 0.053–0.25 mm, ICL and NE had significantly higher N than CNT (Fig. 2). At site 2, ICL had significantly higher N in > 4 mm and 0.25–0.5 mm aggregates than NE and CNT. Whereas at site 3, NE exhibited significantly higher N than ICL and CNT for all aggregate sizes. The only significant difference in soil N at site 4 was observed between oats and grazed pasture mix with mean values of 2.04 and 1.66 g kg^−1^ aggregate, respectively.

**Figure 3:**
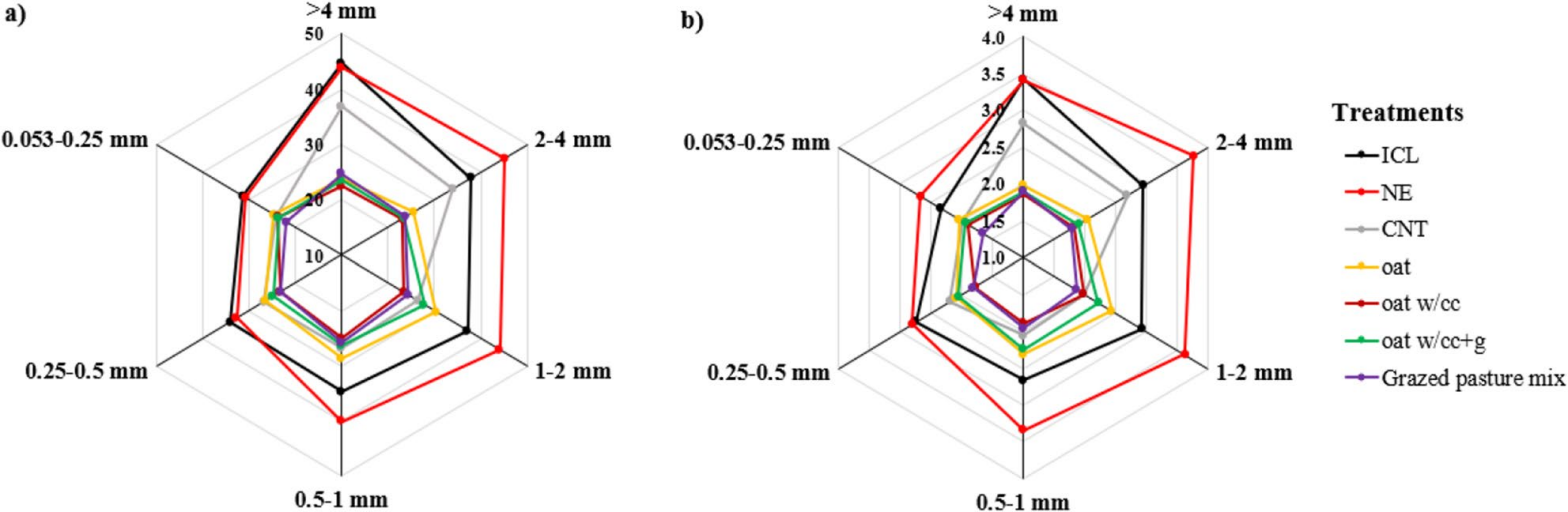
Radar graph depicting the (**a**) aggregate-associated C (g kg^−1^ aggregate) and (**b**) aggregate-associated N (g kg^−1^ aggregate) under different production systems. *ICL* integrated crop–livestock, *NE* natural ecosystem, *CNT* control, *CC* cover crop, *g* grazing.

Overall, ICL and NE systems appeared more stable and favored the greater C and N associated to all aggregate size fractions, followed by CNT, where C and N were enhanced only in > 4 mm and 2–4 mm than the other aggregate sizes (Fig. 3a,b). On the contrary, cropping systems with oats and CC and grazing practices did not depict greater variations in C and N in different aggregate sizes. It was observed that aggregate-associated C and N are following similar trends under different cropping systems, thus also suggesting the tendencies to influence each other positively.

### Soil microbial community composition

Natural ecosystem at site 1 showed significantly (*P* ≤ 0.05) greater total PLFA, total bacterial biomass, gram (+), gram (−) bacteria than CNT, and it did not vary significantly than ICL (Table 3). At site 2, NE showed significantly higher AM fungi and fungi/bacteria ratio than ICL and CNT. Natural ecosystem at sites 2 and 3 resulted in greater total PLFA than ICL, however, it did not vary significantly than CNT. Grazed pasture mix at site 4 depicted a total PLFA of 40.81 nmol g^−1^ soil, which was significantly greater compared to other treatments.

**Table 3:** Soil microbial community structure at 0–5 cm soil depth under different production systems at four different sites.

Treatments	AM fungi	Gram (−)	Gram (+)	Actinomycetes	Fungi/bacteria	Total bacterial biomass	Total PLFA
	nmol g^−1^ soil						
**Site 1**							
ICL^§^	1.44^b†^	16.9^ab^	14.1^ab^	4.84^b^	4.63^b^	30.9^ab^	37.2^ab^
NE	2.45^a^	21.4^a^	16.9^a^	7.91^a^	6.42^a^	38.4^a^	48.8^a^
CNT	1.79^ab^	13.1^b^	10.1^b^	4.40^b^	7.65^a^	23.2^b^	29.4^b^
**Site 2**							
ICL	0.97^b^	11.3^b^	9.02^a^	5.06^a^	4.93^b^	20.3^b^	26.3^b^
NE	2.25^a^	20.9^a^	15.4^a^	8.02^a^	10.80^a^	36.3^a^	48.2^a^
CNT	1.22^b^	13.6^ab^	11.3^a^	5.35^a^	4.71^b^	24.9^ab^	31.5^ab^
**Site 3**							
ICL	1.59^a^	14.3^a^	8.06^b^	6.23^a^	7.13^a^	22.4^b^	30.2^b^
NE	1.57^a^	15.6^a^	14.2^a^	6.22^a^	5.26^a^	29.8^a^	37.5^a^
CNT	1.72^a^	15.9^a^	12.2^a^	4.85^b^	7.93^a^	28.1^a^	35.2^ab^
**Site 4**							
Oat	1.59^a^	13.3^b^	13.2^b^	5.67^c^	5.92^a^	26.6^b^	33.8^b^
Oat w/cc	1.26^a^	14.3^ab^	14.6^ab^	6.13^bc^	4.36^a^	28.9^ab^	36.3^b^
Oat w/cc + g	1.68^a^	13.5^ab^	14.3^ab^	6.79^b^	6.06^a^	27.8^b^	36.3^b^
Grazed pasture mix	1.65^a^	15.6^a^	15.4^a^	8.13^a^	5.29^a^	31.0^a^	40.8^a^
**ANOVA (*P* > *F*)**							
Site 1: Treatments	0.075	0.060	0.066	0.022	0.007	0.062	0.055
Site 2: Treatments	0.049	0.085	0.127	0.196	0.019	0.098	0.083
Site 3: Treatments	0.619	0.335	0.001	0.030	0.283	0.012	0.054
Site 4: Treatments	0.591	0.146	0.065	0.0007	0.441	0.015	0.004

^§^*ICL* integrated crop–livestock, *NE* natural ecosystem, *CNT* control, *CC* cover crop, *g* grazing.

^†^Means followed by different letters within a column under each site are significantly different at *P* ≤ 0.05 according to Fisher’s LSD.

### Principal component analysis

**Figure 4:**
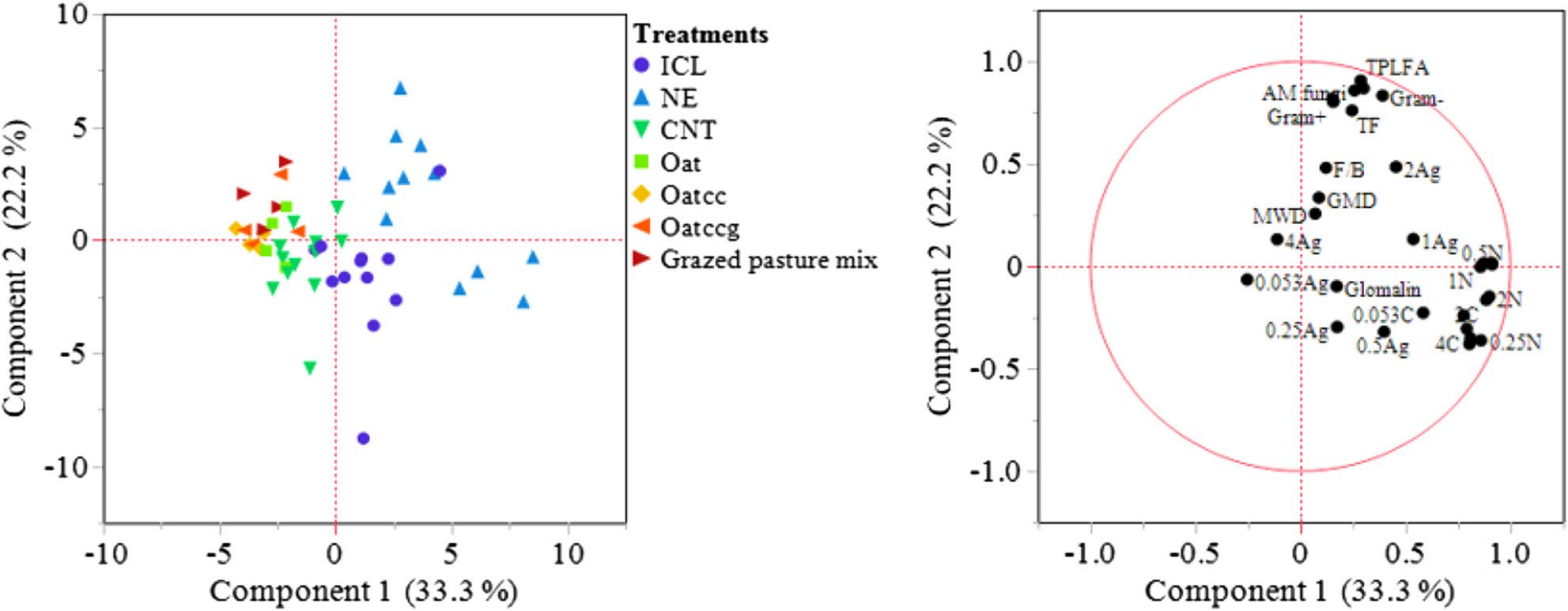
Principal component analysis (PCA) of the soil parameters with scores plotted in the plane of PC1 and PC2 (left) and eigenvectors (right). *ICL* integrated crop–livestock, *NE* natural ecosystem, *CNT* control, *CC* cover crop, *g* grazing, *TPLFA* total phospholipid fatty acid, *AM fungi* arbuscular mycorrhizal fungi, *TF* total fungi, *F/B* fungi/bacteria ratio, *MWD* mean weight diameter, *GMD* geometric mean diameter, *Ag* aggregate size, *C* carbon, *N* nitrogen.

Principal component analysis was performed for measured soil physicochemical and soil biological properties i.e. aggregate size distribution, aggregate-associated SOC and N, glomalin, actinomycetes, gram (+), gram (−), AMF, total bacteria, total fungi, fungi/bacteria, and total PLFA (Fig. 4). The principal components (PC) 1 and 2 explained 33% and 22% of variation, accounting for the total variation of 55%. Different groups formed across the two principal component axes indicated the effect of different management systems on soil atributes. Integrated crop–livestock and NE were the systems with greater C and N fractions associated with different aggregate sizes and enhanced microbial biomass abundance. This represented the major influence of ICL and NE on soil health parameters compared to traditional corn–soybean without grazing or CC and short-term experiment with CC and grazing. Our results showed that soil aggregation and SOC and N in aggregates are related, therefore, may result in aggregate formation and protection of soil organic matter. Soil microbial composition, especially total PLFA, total bacterial and fungal biomass were greater under ICL and NE than CNT, grazed pasture mix, oat + cc, oats + CC + grazing, and oats, indicating that preservation of macroaggregates especially under long-term no-tilled or undisturbed systems positively influences the microbial population.

Pearson's correlation illustrated the positive significant (*P* ≤ 0.05) influence of glomalin on MWD (r = 0.31) and GMD (r = 0.30); and AM fungi on SOC (r = 0.28) and GMD (r = 0.36) (Table 4). Overall, SOC had significant (*P* ≤ 0.05) and positively correlated relationship with soil N and microbial population, e.g., AM fungi, gram (+), gram (−), actinomycetes, fungi/bacteria, total bacterial biomass and total PLFA. It is also noted that increased soil pH can negatively influence SOC and gram (−) bacteria; and higher soil bulk density (BD) can reduce gram (−) bacteria, actinomycetes, total bacterial biomass, and total PLFA. A strong negative correlation between BD and SOC (r = − 0.91) indicated that a reduction in soil C possibly due to increased decomposition can make soil more prone to compaction.

**Table 4:** Pearson’s correlation coefficient between soil physicochemical and biological properties under different productions systems.

Parameters	pH	BD	SOC	N	MWD	GMD	Gloma-lin	AM Fungi	Gram (−)	Gram (+)	Actinomycetes	F/B	TBB	TPLFA
pH		0.58**	**−** 0.53**	0.05	**−** 0.18	**−** 0.13	0.002	**−** 0.10	**−** 0.33*	**−** 0.17	**−** 0.07	0.03	**−** 0.27	**−** 0.23
BD			**−** 0.91**	**−** 0.56**	0.07	0.03	**−** 0.05	**−** 0.22	**−** 0.39*	**−** 0.23	**−** 0.33*	**−** 0.18	**−** 0.34*	**−** 0.36*
SOC				0.69**	**−** 0.04	**−** 0.003	0.02	0.28*	0.40*	0.29*	0.42*	0.29*	0.37*	0.41*
N					**−** 0.19	**−** 0.15	**−** 0.10	0.18	0.22	0.44*	0.52**	0.16	0.34*	0.39*
MWD						0.97**	0.31*	0.27	0.05	0.05	0.12	0.13	0.05	0.07
GMD							0.30*	0.36*	0.10	0.11	0.17	0.21	0.10	0.13
AM Fungi									0.79**	0.61**	0.65**	0.62**	0.75**	0.79**
Gram (−)										0.78**	0.73**	0.37*	0.95**	0.95**
Gram (+)											0.75**	0.05	0.93**	0.91**
Actinomycetes												0.20	0.78**	0.85**
F/B													0.23	0.32*
TBB														0.99**

Values for BD, SOC and N were obtained from ref^31^.

*BD* bulk density, *SOC* soil organic carbon, *N* nitrogen, *MWD* mean weight diameter, *GMD* geometric mean diameter, *F/B* fungi/bacteria, *TBB* total bacterial biomass, *TPLFA* total phospholipid fatty acid.

Bold numbers with ** represent the significant correlation at *P* ≤ 0.0001 and with * represent significant correlation at *P* ≤ 0.05.

## Discussion

### Aggregate size distribution

As hypothesized, the improved soil aggregation was observed under ICL, which is attributed to the presence of animals resulting in higher organic matter contents of total C and N fractions that can significantly enhance soil health over time^32^. Moreover, well-aggregated soils as observed under ICL (> 4 mm) at site 1 and NE (2–4 mm) at site 2 have a greater potential of retaining their structure and may have higher macropores, which facilitate sustained root growth than soils with low aggregation such as under CNT (corn–soybean without grazing or CC) in this study. It also explains the significance of ICL systems with no-tillage and undisturbed grassland, where the formation of stable macroaggregates may occur as a result of incorporation of plant residues, stimulation of root exudates and increased biological activity. Furthermore, it was noticed that ICL system not only enhanced the macroaggregates but accentuated the presence of microaggregates due to persistent binding agents, which are critical in SOC protection against microbial decomposition. When integrating grazing livestock into crop rotation, soil aggregation is typically improved under moderate and controlled grazing than the high intensity grazing systems^33^. Compared to the long-term sites (> 30 years), short-term site 4 did not result in discernible effects of grazing or CC on soil aggregation. However, within this short-term study, grazed pasture mix was able to enhance aggregation of 1–2 and 2–4 mm sized aggregates compared with oats, oats with CC, oats with CC and grazing. To observe the influence of CC and grazing on > 4 mm or < 1 mm aggregate sizes, a longer study period than 3 years might be required.

### MWD, GMD, glomalin-related soil protein

Overall, the observed higher MWD under ICL and NE than CNT showed the bonding of minerals and aggregates due to increased microbial activity and root exudation that improved overall aggregate stability^6^. Higher MWD for integrated systems than continuous corn monoculture has been reported previously^6^. The higher GMD at either ICL or NE system can be explained due to natural reconsolidation of soil than the CNT where soil can be disturbed due to conventional tillage^34^. Integrated crop–livestock at site 1 enhanced glomalin-related soil protein by 1.5 times when compared with CNT. However, this statistical difference (*P* ≤ 0.05) was only observed for 1 out of 3 long-term sites. Similar results were reported in previous study^35^, where glomalin content was not influenced under 15–19 years old grazed pastures at 0–20 cm. Although glomalin serves as an important aggregate binding agent and represents organically-bound soil N in agroecosystems, insignificant results suggest that independent of glomalin, accumulation of soil C can be more important in maintaining stable aggregates under long-term ICL systems.

### Aggregate-associated C and N

Nearly constant C and N concentration was observed in aggregate size classes among soil under ICL and NE at sites 1 and 2, while relatively lower concentration was detected in macro and microaggregates under CNT. Large macroaggregates (> 4 mm) under ICL at site 1 resulted in 1.3–1.5 times significantly higher SOC concentration than NE and CNT. The greater concentration of SOC and N in ICL and NE is attributed to the lack of soil disturbance, crop residue retention, and rhizodeposition, which reduces macroaggregate turnover rate^14^. At site 3, NE enhanced aggregate-associated C and N concentrations and performed significantly better than both ICL and CNT treatments. The higher C and N accrual in the NE than ICL and CNT, especially at site 3, can be due to massive root systems, long-term establishment and absence of cultivation, which contributes to enhanced soil quality, while reducing nutrient vulnerability to loss by oxidation^18,36^. For short-term study at site 4, insignificant differences in aggregate-associated SOC suggested that longer study period of at least > 5 years is required for SOC to respond to grazing and cover crop management. The higher total N under ICL and NE can also be due to the presence of legumes, and brassicas in CC, which are effective at recycling N and may have helped in scavenging N.

An overall increase in C and N cycling under ICL and NE systems has been attributed to ingested pasture being converted into urine and manure. Under these systems, livestock catalyze nutrient cycling by breakdown of complex plant molecules, greater soil incorporation and decomposition of plant residues and soil organic matter, which can maintain or even improve soil fertility by production of organic acids such as fulvic and humic acids^6,8,19^. Moreover, grazing stimulates the carbohydrate exudation from grass roots, which is mostly composed of polysaccharides, a C-O alkyl source^37^. The enhanced C concentration under ICL and NE can also be associated with higher MWD. Integrated system cool-season pasture and winter CC had significantly higher total C and N than the non-integrated continuous corn in previous study^6^. The results from another integrated system study^7^ showed that soybean and oat-Italian ryegrass CC increased total C (1.16 Mg ha^−1^ yr^−1^) and N stocks (0.12 Mg ha^−1^ yr^−1^) under 7 year study period. It is previously reported that ICL system contains labile organic matter pools^10,38^, subsequently showing higher C stocks and greater root densities near soil-surface, which promotes aggregate-associated C stabilization^18,39,40^, higher infiltration rates, thus providing likely benefits to soil function linked to erosion control and soil water relations^41^.

### Soil microbial community composition

Total bacterial biomass, AM fungi, and PLFA were enhanced under NE, which can be result of accumulation of organic residues and higher pasture root mass^7,32^, pasture being grazed can promote exudation of organic compounds by roots, serving as energy sources for microorganisms. The consistent increase in microbial population under NE can also be result of increased SOC and N, however, the same does not hold true for ICL system, where despite observing greater SOC and N, a significant decrease in the microbial population at site 2 was noticed. The enhanced total PLFA under NE system at site 2 is due to concomitant increase in AM fungi, gram (−), fungal/bacterial ratio, and total bacterial biomass compared to ICL. The fungal to bacterial ratio was reduced under ICL compared to NE at sites 1 and 2, pertaining to relatively low abundance of the fungal fatty acid 18:2ɷ6 in grazed system as compared to unmanaged grassland. This finding corroborates the notion that livestock-grazing systems contain bacterial-based decomposition channels and are mostly dominated by gram (+) bacteria and that the fungal population is comparatively more important in decomposer food-webs of native grasslands. These results coincide with previous studies^42,43^. Moreover, the increase in fungal to bacterial ratios under NE system in contrast to ICL at sites 1 and 2 can relate to modifications in soil health with C sequestration, as fungal populations incline towards higher C assimilation proficiencies and greater storage of metabolized C than bacterial populations^9,44^. The grazing intensity also plays a significant role in bacterial and fungal presence. It is previously reported that high grazing intensity had greater bacterial PLFA concentration than the low grazing counterparts in grassland systems^45^. It is considered that under heavily grazed sites in grasslands, bacteria-based energy channels of decomposition dominate other microbial communities, while fungi can successfully enable decomposition in both slightly grazed and non-grazed systems^43^. Grazed pasture mix at short-term study site 4 showed 12–21% higher total PLFA than the oats, oats with CC, oats with CC + grazing systems. It is also possible that this increased total PLFA at site 4 under grazed pasture mix contributed to enhancing the 1–2 and 2–4 mm sized aggregates compared to other treatments. It indicated that though physicochemical properties can take longer (> 8–15 years) in significantly responding to changes in management systems, soil microbial community and structure may show a rapid response (~ 3 years), thus it can be used as an early indicator while assessing the variations in soil health^18,46^.

Overall, NE exemplified the undisturbed grazed mixture with a greater microbial population at sites 1, 2, and 3, when compared to other agricultural systems. Our findings coincide with previous studies where pasture systems performed better than the agricultural soil, in terms of, showing greater microbial biomass and fatty acid signatures related to bacterial and fungal populations, which is mostly attributed to greater surface coverage and absence of tillage practices in pasture systems^9,47,48^. Lower soil microbial communities under ICL system than native Cerrado pasture have been found previously because of reduced soil porosity and macropore continuity resulting in restricted gas diffusion and water movement^18^.

Although the AM fungi abundance was not significant for sites 3 and 4, and significantly lower for ICL system than NE at sites 1 and 2, it should be taken into consideration that FAME analysis cannot reflect species-level changes for fungi and/or bacteria and the variations in microbial community structure for ICL system can be due to changes in abundance and distribution among microbial groups. For example, in a previous study^9^, while increased bacterial population was observed for continuous cotton compared to the ICL system, however, pyrosequencing for bacterial diversity assessment demonstrated disparities between both systems, where greater *Proteobacteria* was seen under ICL system than continuous cotton. Numerous factors such as degree of disturbance, pH level, bulk density, porosity, soil water content, C and N distribution, and residue positioning regulate the amount of bacterial and fungal biomass in agroecosystems^18,49^. Arbuscular mycorrhizal fungi are responsible for formation of macroaggregates (> 0.25 mm) by producing a glycoprotein called glomalin, which is present abundantly in natural and agricultural systems. However, increased grazing intensity, use of excess fertilizers and fungicides can directly or indirectly reduce mycorrhizal population by influencing soil organisms accountable for converting soil organic matter into plant nutrients^38^. Animals may also cause moderate soil compaction affecting the fungal biodiversity and soil pore space^6,38^.

### Relationship among measured soil properties

Based on PCA, it is derived that integrated crop–livestock and natural ecosystem of native grassland can provide substrate for the microbial composition and enhance aggregate-associated C and N fractions. A positive correlation between SOC and microbial communities suggested the inclination of microbes to affect SOC and N turnover and vice-versa through interaction with crop–livestock grazing, vegetation, and soil properties. Fungi exhibited insignificant responses to changes in soil pH and bulk density than bacteria because chitinous cell walls make fungi more resistant and resilient to variations in soil conditions^50,51^. A reduction in gram (−) bacteria may indicate the presence of stressed soil conditions due to pH and increased bulk density, which has previously been observed in other studies^52,53^. A significant negative correlation between bulk density and SOC, N, gram (−), actinomycetes, total bacteria, and total PLFA indicated that the microorganisms influenced the soil compaction and related SOC and N. Moreover, positive correlation between SOC and microbial composition suggested that microbes can influence C sequestration in the soil via a shift in community structure. Microbial composition is influenced by soil C, whereas N is a critical biogenic element that improves microbial growth and their ability to utilize soil C^54^.

## Materials and methods

### Experimental site and treatments

The study was conducted in South Dakota at four locations consisting of three on-farm sites: site 1 (Salem, SD); site 2 (Bristol, SD); site 3 (Bristol, SD); and one experimental site 4 (Beresford, SD). The study sites have Köppen climate classification of warm humid continental weather with mean annual temperature of 6 °C and annual precipitation of 710 mm. Detailed site and treatment description is presented in Table 5. Sites 1, 2, and 3 consisted of similar long-term (> 30-year) experiments of: (i) integrated crop–livestock (ICL) system corn–soybean–oats (*Avena sativa* L.)-CC with cattle grazing, (ii) natural ecosystem (NE) as grazed native pasture, and (iii) corn–soybean without grazing or CC as control (CNT). Site 4 is an experimental site with study duration of 3-years consisting of treatments: (i) oats, (ii) oats with CC, (iii) oats with CC + grazing, and (iv) grazed pasture mix. Natural ecosystem was present at producers’ locations for about > 50 years and part of this area was converted to croplands (ICL and CNT) by the producer. Natural ecosystem was used as a reference to compare any improvement that may occur in soil properties under ICL or CNT systems. The field size was 40 ha, with ICL and CNT treatments located within 50-m distance of each other, and natural ecosystem area was located 100–200 m away from the study sites. From November to March, a group of Aberdeen Angus cattle (*Bos taurus*) were used for grazing of corn and soybean residues, and CC every year^31^. Pastures were grazed during May and October. The experimental site 4 was set up as a randomized complete block design with four replications of plot size 18 m × 36 m. From this site, four soil samples were collected from each plot and were composited for soil analyses. At producers’ sites, three pseudo-replicates were established depending on the landscape position and soil characteristics. Four soil samples were taken from each established pseudo-replicate. Cover crop treatment consists of legumes, grasses, and brassicas such as pea, sorghum, radish, lentil, cowpea, turnip, and oats at four sites. The study complies with local and national regulations. All methods were carried out in accordance with relevant guidelines and regulations and all experimental protocols were approved by the Institutional Animal Care and Use Committee of South Dakota State University. Seeds used in the study were supplied by Millborn Seeds Inc (Brookings, SD) and are property of South Dakota State University.

**Table 5:** Detailed description of soil type, treatments, and management for the study sites.

Location	Soil type	Treatments	Seed rates	Fertilizer application
**Site 1-Salem, SD** 43° 44′ 42.0″ N 97° 17′ 38.4″ W	Davison soil series (fine-loamy, mixed, superactive, mesic Aeric Calciaquolls)	1. ICL^†^: corn–soybean–oats–CC with cattle grazing 2. NE: Natural ecosystem 3. CNT: corn–soybean without grazing	Corn: 79,074 seeds ha^−1^ Soybean: 284,171 seeds ha^−1^ Oats: 2,471,052 seeds ha^−1^ CC: 27 kg ha^−1^	Corn: 168 kg N ha^−1^, 73 kg P ha^−1^, 56 kg K ha^−1^ Soybean: 73 kg P ha^−1^, 67 kg K ha^−1^ Oats: 67 kg N ha^−1^, 56 kg P ha^−1^, 56 kg K ha^−1^
**Site 2-Bristol, SD** 45° 16′ 24.55″ N 97° 50′ 13.34″ W	Poinsett-Forman complex (fine-silty, mixed, superactive, frigid Calcic Hapludolls)	1. ICLS: corn–soybean–CC with cattle grazing 2. NE: Natural ecosystem 3. CNT: corn–soybean without grazing	Corn: 75,000 seeds ha^−1^ Soybean: 475,000 seeds ha^−1^ CC: 425,000 seeds ha^−1^	Soybean: 56 kg MAP ha^−1^
**Site 3-Bristol, SD** 45° 16′ 24.55″ N 97° 50′ 13.34″ W	Forman-Aastad loams (fine loamy, mixed, frigid Udic Argiborolls)	Similar as site 2	Corn: 81,250 seeds ha^−1^ Soybean: 445,000 seeds ha^−1^ CC: 23 kg ha^−1^	223 kg MAP ha^−1^ 125 kg K ha^−1^
**Site 4-Beresford, SD** 43° 03′ 00″ N 96° 53′ 49″ W	Egan-Trent (fine-silty, mixed, superactive, mesic Pachic Haplustolls)	1. Oats 2. Oats with CC 3. Oats with CC and grazing 4. Grazed pasture mix	Corn: 80,000 seeds ha^−1^ Soybean: 350,000 seeds ha^−1^ Oats: 90 kg ha^−1^ CC: 17 kg ha^−1^	Corn: 118 kg N ha^−1^ Oats: 74 kg N ha^−1^

CC is a mixture of grasses, legumes, and brassicas: pea, sorghum, radish, lentil, cowpea, turnip, and oats.

^†^*ICL* integrated crop–livestock, *NE* natural ecosystem, *CNT* control, *CC* cover crop, *g* grazing.

### Soil sampling and measurements

#### Aggregate size distribution

Soil samples were collected during summer 2019 at 0–5 cm depth using a push probe auger. The wet sieving method of aggregate separation was performed as suggested by other studies^55^, and^56^. Air-died soil (100 g) was submerged in wet sieving vertical shaker apparatus (CSC sieve shaker, Fairfax, VA) adjusted to 90 rpm and 1.3 cm stroke length for 5 min on top of a set of 4, 2, 1, 0.5, 0.25-, and 0.053-mm sieves. Aggregate size distribution was determined by soil remained on each sieve and further dried at 105 °C oven temperature, weighed and expressed as a fraction of the initial mass of samples used.

The mean weight diameter (MWD) was calculated using the following equation^57^:

$$MWD= \sum_{i=1}^{n}xiwi$$

where *xi* is the mean diameter of aggregate size on each sieve (mm), *wi* is aggregate mass retained on each sieve (g) as a fraction of total dry weight of soil (g), and *n* is the number of aggregate size fractions.

The geometric mean diameter (GMD) was calculated by the equation^58,59^:

$$GMD =exp\left[\sum_{i=1}^{n}wi\,{\mathrm{log}}\,xi/\sum_{i=1}^{n}wi\right]$$

where *xi* is the mean diameter of each size fraction (mm) and *wi* is the fraction of total soil sample weight present in the size fraction *i*.

### Aggregate-associated C and N

Aggregate size fraction retained on each sieve was ground and a 0.250-g soil sample was weighed in a tin foil cup for analyzing SOC and N in aggregates with a LECO TruSpec Analyzer (LECO Corporation, St. Joseph, MI) using the dry combustion method.

### Phospholipid fatty acid (PLFA) analysis

#### Extraction

To assess microbial community structure, total soil lipids were extracted by shaking approx. 1–2 g of soil in 4 ml of Blight & Dyer reagent (200 ml 50 mM K_2_HPO_4_ buffer in deionized H_2_O, 500 ml methanol, 250 ml chloroform) and 19:1 phosphatidylcholine (Avanti Polar Lipids, US) internal PLFA standard followed by sonication at room temperature. Samples were centrifuged in the 5804 R centrifuge (Eppendorf, US) at 4000 rpm for 15 min to separate solid and liquid phases. Supernatant was added with 1 ml of each deionized water and chloroform and centrifuged again at 4000 rpm for 15 min. Separated liquid phase was placed in a SpeedVac™ vacuum concentrator (Thermo Scientific, US) for drying at low/ambient temperature for 1 h.

#### Lipid separation

The samples were dissolved with 1 ml chloroform and transferred to conditioned HyperSep™ solid-phase extraction (SPE) columns (Thermo Scientific, US), containing 50 mg silica per 1 ml column and allowed to gravity drain. A 1.5 ml clean glass catch vial was placed below each column and phospholipids were eluted using 0.5 ml of the 5:5:1 chromatography eluent solution (methanol: chloroform: deionized water) to the SPE columns. The collected solution was dried in a SpeedVac™ vacuum concentrator for approx. 1 h at ambient temperature.

#### Trans-esterification

A 0.2 ml of trans-esterification reagent was added to the dried samples followed by incubation at 37℃ for 15 min. A 0.4 ml of 0.075 M acetic acid and 0.5 ml of chloroform was added to each tube and bottom phase after vortex was transferred to a GC vial followed by drying in SpeedVac™ vacuum concentrator for 20–30 min at ambient temperature. The samples were further resuspended using 100 µl of hexane and analyzed using an Agilent 2030-GC equipped with a CP-7693 auto-sampler and a flame ionization detector (FID). Fatty acid peaks were identified by comparing the retention times to MIDI PLFAD2 calibration mix using SHERLOCK software v.6.2 (MIDI Inc, US). Fatty acids were used as functional group signatures for various microorganisms and each PLFA was expressed as nmol g^−1^ soil. The identified bacteria biomarkers included ten gram (+) (15:1 iso ω6c, 15:0 iso, 15:0 anteiso, 16:0 iso, 17:1 iso ω9c, 17:0 iso, 17:0 anteiso, 18:0 iso, 18:1 ω9c, 20:00), ten gram (−) (16:1 ω9c, 16:1 ω7c, 17:1 ω8c, 17:0 cyclo ω7c, 16:0 2OH, 18:1 ω7c, 18:1 ω5c, 19:0 cyclo ω7c, 20:1 ω9c, 21:1 ω9c), four actinomycetes (16:0 10-methyl, 17:1 ω7c 10-methyl, 18:1 ω7c 10-methyl, 18:0 10-methyl), AM fungi (16:1 ω5c), and fungi (18:2 ω6c) biomarkers.

### Glomalin-related soil protein

Soil protein was extracted using the method provided by^60^ with some modifications by^61^. Air-dried soil (3 g) was weighed into a glass tube and 24 ml of 20 mM sodium citrate buffer was added, followed by shaking at 180 rpm for 5 min. The tubes containing samples were autoclaved at 121 °C, 15 psi for 30 min and cooled. Two-ml of the slurry was retracted to a microcentrifuge tube and soil particles were separated by centrifuging the slurry at 10,000 × gravity. Two-ml of Thermo Pierce™ bicinchoninic acid (BCA) protein assay solution was added to 0.1 ml of clarified extract and was incubated for 1 h at 60 °C. Color development was read using BioTek spectrophotometric plate reader. Extractable soil protein content was determined by multiplying the extract’s protein concentration by the extractant’s volume and dividing by soil (g) used.

### Statistical analyses

The SAS 9.4 version (SAS Institute, Cary, North Carolina, USA) was used with one-way ANOVA procedure to analyze the influence of different crop management systems on soil physical and biochemical parameters. Each site was analyzed separately where ICL, NE, and CNT treatments were considered as fixed effects. Fisher’s protected least significant difference (LSD) was used for mean separation of treatments at a significance level of 0.05. Shapiro–Wilk’s test was used to analyze the normality of the data and Levene’s test was used to assess the homogeneity of variances. Principal component analysis (PCA) of the full dataset was analyzed using the PRINCOMP procedure of SAS 9.4. Pearson’s correlation was performed using different soil physicochemical properties and soil microbial structure using PROC CORR statement of SAS version 9.4. The PCA was used to subgroup experimental treatments based on the measured soil parameters by generating the eigenvectors of these parameters called loadings and principal component (PC) scores for each unit. Each eigenvector loading indicates the direction and magnitude of association between soil properties and treatments^62^.

## Conclusion

This study evaluated the influence of integrated crop–livestock management, cover crops, and grazing compared to the traditional corn–soybean rotation on selected soil health indicators focusing mainly on aggregate size distribution, MWD, GMD, glomalin-related soil protein, aggregate-associated C and N, and soil microbial community structure. The performance of these measured soil parameters under integrated crop–livestock system was also compared with > 50 years old native grassland used as a reference. The overall significantly greater performance was observed in the soil parameters at sites 1, 2, and 3, indicating that the long-term establishment of producers’ sites for > 30 years was efficient and showed considerable differences in soil health contrasting to the experimental site which had been established for a comparatively shorter duration of 3 years. In particular, ICL resulted in improved aggregate fraction with greater > 4 mm and 0.053–0.25 mm aggregates compared with NE and CNT, attributed to animal presence, incorporated manure, and improved microbial diversity. Insignificant differences in glomalin protein at sites 2, 3, and 4 among all treatments represented that independent of glomalin, soil C could play an important role in binding aggregates. Improved C and N cycling was observed not only in macroaggregates but also in microaggregates under ICL at site 1 and both ICL and NE at site 2 when compared with corn–soybean control. Natural ecosystem performed well in terms of increasing total PLFA at all three producers’ sites, which can be due to higher vegetation cover, higher pasture root mass, absence of cultivation, hence higher root-associated organic compounds enhanced due to grazing. Phospholipid fatty acid analysis showed that ICL and NE at site 1 had higher total PLFA, total bacterial biomass, gram (+), gram (−) bacteria than corn–soybean rotation, suggesting the improvement in the soil microbial structure by the addition of livestock into the crop production system. The response of grazed pasture mix under short-term experiment (~ 3 years) was inconspicuous in terms of variations in aggregate-associated C and N, however, it did increase the 1–2 and 2–4 mm aggregate distribution compared with the oats/CC/grazing practices, possibly due to observed higher total PLFA. The observed significant differences in aggregation and microbial composition at the experimental site 4 suggest that these may serve as physical and biological indicators for short-term experiments and can better detect changes in soil than C and N variations as a result of different management systems. Integrated crop–livestock system may have similar or greater benefits as a long-term natural ecosystem in terms of increasing macroaggregate and microaggregate fraction along with associated C and N cycling, total PLFA, total bacterial biomass, gram (+), gram (−) bacteria compared to traditional corn–soybean without grazing or cover crops.
